# Correlation of Serum Growth Differentiation Factor 15 With Metabolic Syndrome and Its Components in Patients With Type 2 Diabetes Mellitus

**DOI:** 10.1155/jdr/5345541

**Published:** 2025-12-10

**Authors:** Yiran Zhao, Yonghong Cao, Yue Li, Xinxiu Zhang, Wu Dai, Jiajia Song, Dechao Yin, Xiaofang Han

**Affiliations:** ^1^ Department of Endocrinology, The Second People′s Hospital of Hefei, Hefei Hospital Affiliated to Anhui Medical University, Hefei, China, ahmu.edu.cn; ^2^ The Fifth Clinical College of Anhui Medical University, Hefei, China

**Keywords:** growth differentiation factor 15, metabolic syndrome, obesity, Type 2 diabetes mellitus

## Abstract

**Purpose:**

The purpose of this study is to explore the association between serum growth differentiation factor 15 (GDF15) and metabolic syndrome (MS), as well as its components in patients with Type 2 diabetes mellitus (T2DM).

**Patients and Methods:**

Data on 125 patients with T2DM admitted to the Department of Endocrinology of the Second People′s Hospital of Hefei City between August 2022 and June 2023 were retrospectively collected. According to the diagnostic criteria of MS, the patients were divided into two groups: T2DM alone (67 cases) and T2DM combined with MS (58 cases). General conditions and laboratory indicators of the patients were collected to analyze the correlation of GDF15 expression in T2DM and combined MS.

**Results:**

The GDF15 level in the T2DM with MS group was higher than that in the T2DM group (*p* < 0.001); specifically, the median (interquartile range) of GDF15 in the T2DM with MS group was 494.86 (355.72, 925.77) pg/mL, compared with 266.47 (174.49, 405.09) pg/mL in the T2DM alone group. Serum GDF15 was used as a grouping variable, and the prevalence of MS increased as GDF15 levels increased. Correlation analysis showed that GDF15 was found to be positively correlated (*p* < 0.05) with systolic blood pressure (*r* = 0.274), body mass index (*r* = 0.239), uric acid (*r* = 0.182), triglycerides (*r* = 0.314), and MS prevalence (*r* = 0.506). Binary logistic regression results showed that GDF15 was a statistically independent risk factor for T2DM combined with MS (*p* < 0.05). The diagnostic value of GDF15 for T2DM with MS was validated by ROC curve analysis (AUC [95*%*CI] = 0.793 [0.716–0.870], *p* < 0.001) with an optimal cut‐off value of 395.81 pg/mL, a sensitivity of 71%, and a specificity of 73%.

**Conclusion:**

Serum GDF15 is strongly correlated with T2DM combined with MS and its components, and elevated GDF15 is valuable for the diagnosis of T2DM combined with MS.

## 1. Introduction

Diabetes mellitus (DM) affects 400 million people globally and is a serious public health issue [1]. Numerous long‐term microvascular and macrovascular problems may arise as the condition worsens. Retinopathy, cataracts, nephropathy, and neuropathy are instances of microvascular repercussions; stroke, cardiovascular disease, coronary artery disease, cerebrovascular disease, and diabetic foot are examples of macrovascular issues. In 2015, there were around 415 million people with diabetes worldwide, more than 90% of whom had type 2 diabetes mellitus (T2DM). By 2040, that number is predicted to grow to 640 million, or 642 million people [2]. In actual clinical settings, there is a 70.0%–80.0% chance that a patient with Type 2 diabetes also has a concomitant metabolic syndrome (MS) [3]. T2DM combined with MS increases the risk of developing heart disease or stroke‐related cardiovascular disease [4]. Early detection of T2DM in conjunction with MS and the execution of therapies thus assumes a crucial role.

Despite the complexity of the MS′s pathogenic avenues, insulin resistance (IR) is considered to be the central component of the disease′s etiology [5]. In Type 2 diabetes, IR is a major pathogenic mechanism [6]. Growth differentiation factor 15 (GDF15), also known as macrophage inhibitory cytokine, was discovered in a macrophage lineage clone in 1997. It belongs to the superfamily of transforming growth factor *β* (TGF*β*) [7, 8]. By modulating the metabolic activity of lipolytic genes, GDF15 is hypothesized to have diagnostic and therapeutic value in the treatment of IR, T2DM, obesity, and glycolipid metabolism [9]. Serum GDF15 has been linked in recent research to the development of cancer and cardiovascular disease; however, there are still fewer studies examining the connection between serum GDF15 and T2DM in combination with MS, and the clinical application has not yet been promoted. Therefore, this study explored the association between GDF15 and MS as well as its components in T2DM patients.

## 2. Materials and Methods

### 2.1. Study Design

A retrospective collection of 125 T2DM patients who were admitted to the Second People′s Hospital of Hefei City between August 2022 and June 2023 was made. The study participants were split into two groups: the T2DM group (*n* = 67) and the T2DM combined with the MS group (*n* = 58). Inclusion criteria were as follows: every research participant satisfied the 1999 WHO diagnostic standards for diabetic mellitus [10]. Exclusion criteria were as follows: (1) other types of DM; (2) severe infections and serious diseases of the heart, kidney, liver, psychiatric disorders, tumors, and so on; (3) pregnant and lactating women; (4) those with acute complications of DM; (5) other endocrine disorders, such as secondary hypertension, hypothyroidism or hyperthyroidism, and so on; (6) major traumatic injuries, surgeries, infections, and other stressful events in the last 3 months; and (7) incomplete data.

## 3. Methodologies

1. Baseline data and laboratory test results were registered: (1) Baseline data: Gender, age, duration of DM, height, weight, systolic blood pressure (SBP) and diastolic blood pressure (DBP), and blood pressure were taken as the average of three measurements taken in a quiet state. (2) Collection and measurement of peripheral blood specimens: Venous blood was collected early in the morning after 8 h of fasting, and various routine blood and biochemical indexes were measured: serum creatinine (Scr), uric acid (UA), triglyceride (TG), cholesterol (CHOL), C‐reactive protein (CRP), low‐density lipoprotein cholesterol (LDL‐C), high‐density lipoprotein cholesterol (HDL‐C), alanine transaminase (ALT), aspartate aminotransferase (AST), glycosylated hemoglobin A1c (HbA1c), fasting blood glucose (FBG), and fasting insulin (FINS). Serum GDF15 concentration was measured by ELISA. Body mass index (BMI): BMI = weight (kg)/height squared (m^2^); homeostasis model assessment‐insulin resistance (HOMA‐IR): HOMA‐IR = [FPG (mmol/L) × FINS (mU/L)]/22.5.
2. Diagnostic criteria for MS: Referring to the 2009 joint interim statement by the International Diabetes Federation Task Force on Epidemiology and Prevention; National Heart, Lung, and Blood Institute; American Heart Association; and so on [11], MS is diagnosed if three or more of the following criteria are met: (1) central obesity: waist circumference ≥ 90 cm for men or ≥ 85 cm for Chinese women (adjusted for ethnicity); (2) hyperglycemia: FBG ≥ 6.1 mmol/L, 2‐h postprandial glucose ≥ 7.8 mmol/L, or previously diagnosed and treated diabetes; (3) hypertension: SBP/DBP ≥ 140/90 mmHg or previously diagnosed and treated hypertension; (4) hypertriglyceridemia: fasting TG ≥ 1.7 mmol/L, or receiving treatment; and (5) low HDL‐C: < 1.03 mmol/L in men or < 1.29 mmol/L in women, or receiving treatment. Participants who did not fit the MS diagnostic criteria were assigned to the T2DM group (also known as the T2DM group), whereas those who did fit the MS diagnostic criteria were assigned to the T2DM combined MS group (also known as the T2DM combined MS group).

### 3.1. Statistic Analysis

Statistical analysis was performed using SPSS 26.0. Normally distributed quantitative variables were reported as mean ± standard deviation (−*x* ± *s*), with one‐way analysis of variance (ANOVA) for comparisons between multiple groups and *t*‐test for comparisons between two groups. Measurements that were not normally distributed were reported as median and interquartile spacing (M[P25, P75]), and within‐group comparisons were made using the Mann–Whitney *U* test or the Kruskal–Wallis *H* test. Categorical variables were reported as the number of cases (percentage) (*n* [percentage]), and comparisons between groups were performed using the *χ* ^2^ test. Variables related to GDF15 were analyzed using the Pearson and Spearman correlation analysis, while binary logistic regression was used to study risk factors associated with MS. Plotting the subject characteristic curve (ROC) allowed researchers to examine the diagnostic utility of related indicators for MS. *p* < 0.05 indicates a statistically significant distinction.

## 4. Results

### 4.1. Subject Characteristics

The clinical data across the patient groups showed that patients in the T2DM combined with MS group had decreased levels of HDL‐C and elevated levels of SBP, DBP, BMI, UA, TG, HOMA‐IR, and GDF15 than patients in the T2DM group (Table 1). This difference was statistically significant (*p* < 0.05).

**Table 1:** Comparison of clinical data of patients with and without comorbid metabolic syndrome.

**Groups**	**T2DM group (** **n** = 67**)**	**T2DM + MS group (** **n** = 58**)**	**χ** ^2^ **/t/z**	**p** **value**
Age (years old)	61.24 ± 10.45	58.40 ± 12.34	1.397	0.165
Sex (male/female)	34/33	23/35	1.542	0.214
Duration of disease (years)	8 (3, 14)	7 (2, 11)	−0.977	0.328
SBP (mmHg)	126 (118, 139)	142 (128.5, 149.0)	−3.893	< 0.001
DBP (mmHg)	77 (71, 80)	82 (72.00, 90.25)	−2.715	0.007
BMI (kg/m^2^)	23.34 (21.87, 24.45)	26.49 (25.08, 27.92)	−7.166	< 0.001
UA (*μ*mol/L)	290.131 ± 79.03	338.66 ± 95.99	−3.099	0.002
Scr (*μ*mol/L)	60 (47, 70)	64 (54.28, 75.18)	−1.215	0.224
TG (mmol/L)	1.16 (0.82, 1.60)	1.96 (1.28, 2.73)	−4.963	< 0.001
CHOL (mmol/L)	4.58 ± 1.06	4.40 ± 1.23	0.874	0.384
HDL‐C (mmol/L)	1.25 (1.10, 1.43)	1.07 (0.90, 1.24)	−3.790	< 0.001
LDL‐C (mmol/L)	2.46 ± 0.85	2.53 ± 0.96	−0.483	0.630
CRP (mg/L)	2 (2.0, 2.7)	2 (2.0, 3.5)	−0.668	0.504
HbA1c (%)	8.5 (7.2, 10.5)	8.25 (7.38, 10.50)	−0.277	0.782
FBG (mmol/L)	7.59 (6.42, 9.50)	8.05 (6.22, 9.40)	−0.805	0.421
HOMA‐IR	1.41 (0.83, 2.37)	2.90 (1.97, 4.85)	−4.859	< 0.001
GDF15 (pg/mL)	266.47 (174.49, 405.09)	494.86 (355.72, 925.77)	−5.636	< 0.001

Note: Data are presented as mean ± SD, median (interquartile range), or number (percentage). Student′s *t*‐test and Mann–Whitney *U* test were used for comparisons between the two groups. Chi‐squared test was used for between‐group comparisons of count data.

Abbreviations: BMI, body mass index; CHOL, cholesterol; CRP, C‐reactive protein; DBP, diastolic blood pressure; FBG, fasting blood glucose; GDF15, growth differentiation factor 15; HbA1c, glycosylated hemoglobin; HDL‐C, high‐density lipoprotein cholesterol; HOMA‐IR, insulin resistance index; LDL‐C, low‐density lipoprotein cholesterol; MS, metabolic syndrome; SBP, systolic blood pressure; Scr, blood creatinine; T2DM, Type 2 diabetes mellitus; TG, triglyceride; UA, uric acid.

### 4.2. Comparison of Clinical Data With Different GDF15 Levels

Serum GDF15 was used as a grouping variable and was divided into Groups A, B, C, and D depending on the quartiles of GDF15 levels. It could be found that the number of MS prevalence cases in each group was 3, 14, 17, and 24, accounting for 9.4%, 45.2%, 54.8%, and 77.4%, respectively, and with the elevation of GDF15 levels, the MS prevalence gradually increased with increasing GDF15 levels (*p* < 0.05). Additionally, there was a gradual rise in GDF15 levels accompanied by elevated SBP and TG levels (*p* < 0.05, Table 2). HOMA‐IR displayed a trend of steady increase with the elevation of GDF15, but it did not achieve a statistically significant difference.

**Table 2:** Comparison of clinical data for different GDF15 levels.

**Groups**	**Group A (** **n** = 32**)**	**Group B (** **n** = 31**)**	**Group C (** **n** = 31**)**	**Group D (** **n** = 31**)**	**χ** ^2^ **/z/F**	**p** **value**
Age (years old)	60.13 ± 11.24	61.13 ± 10.34	60.03 ± 12.16	57.39 ± 11.89	0.725	0.539
Sex (male/female)	18/14	21/10	14/17	16/15	3.411	0.333
Duration of disease (years)	6.5 (1, 11.75)	4 (2, 11)	10 (5, 15)	8 (2, 13)	2.979	0.395
SBP (mmHg)	125.94 ± 17.10	132.90 ± 15.87	135.23 ± 15.67	142.06 ± 23.65	4.136	0.008
DBP (mmHg)	77.5 (72.5, 81.5)	78 (70, 89)	76 (70, 92)	80 (70, 92)	1.719	0.633
BMI (kg/m^2^)	23.75 (22.06, 24.76)	25.35 (23.44, 26.73)	24.44 (22.04, 27.47)	25.80 (24.00, 27.11)	11.653	0.009
UA (*μ*mol/L)	270.2 (224.45, 331.75)	296 (259.1, 402)	283 (223, 363)	346 (309, 391)	7.690	0.053
Scr (*μ*mol/L)	66 (45.18, 74.38)	64 (56, 76)	59 (44, 66.2)	63.5 (55, 73)	3.258	0.354
TG (mmol/L)	1.21 (0.80, 1.60)	1.35 (1.02, 1.98)	1.34 (1.16, 2.08)	1.94 (1.18, 3.26)	11.812	0.008
CHOL (mmol/L)	4.28 ± 0.96	4.53 ± 1.06	4.68 ± 1.00	4.49 ± 1.48	0.652	0.583
HDL‐C (mmol/L)	1.2 (1.05, 1.36)	1.16 (0.97, 1.34)	1.17 (1.02, 1.31)	1.18 (0.89, 1.35)	1.186	0.756
LDL‐C (mmol/L)	2.29 (1.60, 3.15)	2.44 (1.84, 3.04)	2.63 (2.10, 3.09)	2.09 (1.78, 3.22)	2.408	0.492
CRP (mg/L)	2 (2.00, 2.45)	2 (2.0, 2.3)	2.2 (2.0, 3.9)	2 (2.0, 3.5)	2.329	0.507
HbA1c (%)	8.15 (6.83, 9.50)	8.2 (7.3, 9.9)	9.2 (7.5, 11.7)	8.9 (7.8, 10.5)	6.154	0.104
FBG (mmol/L)	7.72 (6.20, 9.78)	7.4 (5.91, 8.60)	7.8 (7.30, 9.55)	8.14 (6.10, 9.40)	2.736	0.434
HOMA‐IR	1.80 (0.94, 2.87)	1.93 (1.08, 3.43)	2.23 (0.75, 4.02)	2.46 (1.41, 3.69)	3.088	0.378
MS (%)	3 (9.4%)	14 (45.2%)	17 (54.8%)	24 (77.4%)	30.539	< 0.001

*Note:* Data are expressed as mean ± standard deviation, median (interquartile range), or number (percentage). One‐way ANOVA and the Kruskal–Wallis *H* test were used to compare samples from three or more groups. The chi‐square test was used for between‐group comparisons of count data.

Abbreviations: BMI, body mass index; CHOL, cholesterol; CRP, C‐reactive protein; DBP, diastolic blood pressure; FBG, fasting blood glucose; GDF15, growth differentiation factor 15; HbA1c, glycosylated hemoglobin; HDL‐C, high‐density lipoprotein cholesterol; HOMA‐IR, insulin resistance index; LDL‐C, low‐density lipoprotein cholesterol; SBP, systolic blood pressure; Scr, blood creatinine; TG, triglyceride; UA, uric acid.

### 4.3. Correlation Analysis of GDF15 Level and MS‐Related Indexes

Further analysis into the relationship between GDF15 and MS‐related indices showed that GDF15 had a positive correlation with UA (*r* = 0.182), TG (*r* = 0.314), SBP (*r* = 0.274), BMI (*r* = 0.239), and the prevalence of MS (*r* = 0.506) (*p* < 0.05). Additionally, GDF15 levels were higher in the MS group than in the non‐MS group (Table 3 and Figure 1).

**Table 3:** Correlation analysis of GDF15 with MS and its components.

**Variables**	**r**	**p** **value**
The prevalence of MS	0.506	< 0.001
SBP	0.274	0.002
BMI	0.239	0.007
UA	0.182	0.042
TG	0.314	< 0.05

*Note:* GDF15 was positively correlated with MS prevalence and its components (SBP, BMI, UA, and TG) (*p* < 0.05).

Figure 1Correlation analysis between GDF15 and MS‐related indicators. Note: (a) Correlation between GDF15 and SBP (*r* = 0.274, *p* < 0.05). (b) Correlation between GDF15 and BMI (*r* = 0.239, *p* < 0.05). (c) Correlation between GDF15 and UA (*r* = 0.182, *p* < 0.05). (d) Correlation between GDF15 and TG (*r* = 0.314, *p* < 0.05).

**Figure:**
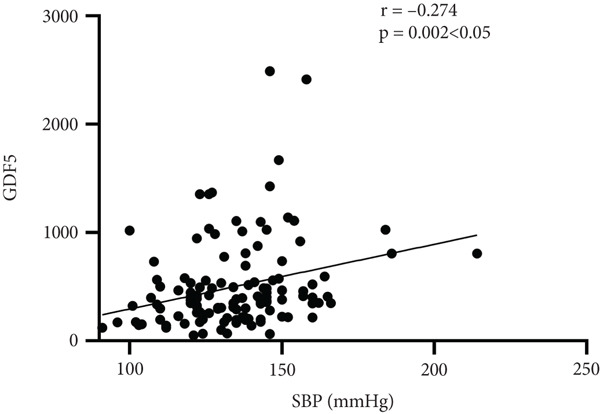
(a)

**Figure:**
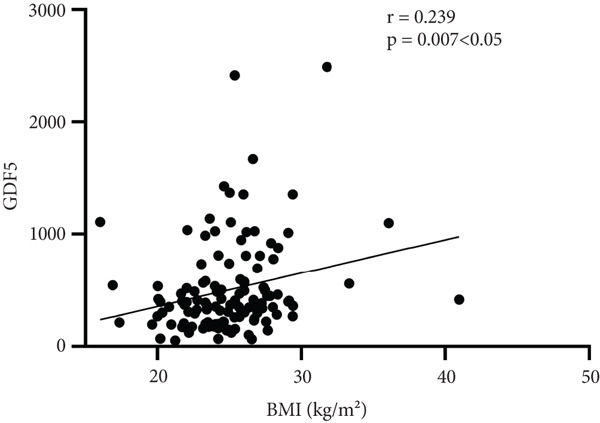
(b)

**Figure:**
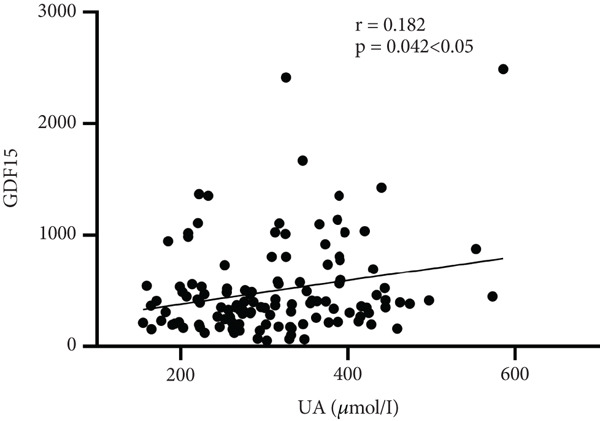
(c)

**Figure:**
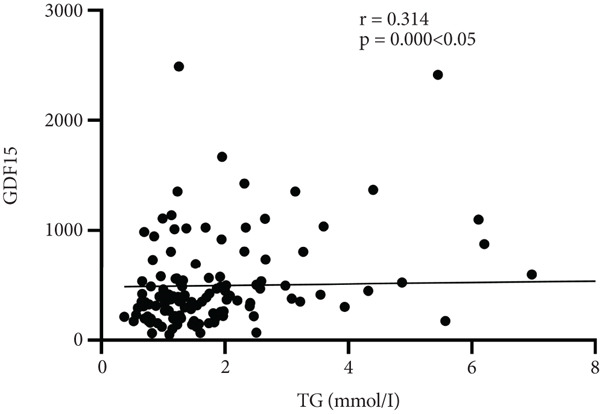
(d)

### 4.4. Binary Logistic Regression Analysis of Risk Factors for the Occurrence of T2DM Combined With MS

Because GDF15 significantly deviated from the normal distribution, we carried out the ln transformation preprocessing in the logistic regression. SBP, DBP, BMI, UA, TG, HOMA‐IR, and GDF15 were the risk factors for the development of MS in combination with T2DM, based on the findings of univariate logistic regression analysis utilizing the combination of the MS (*yes* = 1, *no* = 0) as the dependent variable and the statistically significant indexes in Table 1 as the independent variables (*p* < 0.05, Table 4). In binary logistic regression analysis controlling for confounders, in the crude model, for every one standard deviation change in lnGDF15, the incidence of MS in T2DM patients increased 6.856‐fold (95% CI: 3.180–14.781, *p* < 0.05), and with the inclusion of the composite variables of sex, SBP, and DBP (Model 1), for every one standard deviation change in lnGDF15, the likelihood of having MS in T2DM patients rose 6.697 times (95% CI: 2.898–15.473, *p* < 0.001). After continuing to add the composite variables of age, BMI, UA, TG, HDL‐C, and HOMA‐IR to Model 1 (Model 2), the risk of MS in T2DM patients increased 6.649‐fold (95% CI: 2.224–19.885, *p* < 0.001), and the results demonstrated that GDF15 was an independent risk factor for MS in the combination with T2DM (*p* < 0.05, Table 5).

**Table 4:** Univariate logistic regression analysis of factors affecting metabolic syndrome in T2DM patients.

**Characteristics**	**B**	**SE**	**p** **value**	**OR**	**95% CI**
SBP (mmHg)	0.043	0.012	< 0.001	1.044	1.019–1.068
DBP (mmHg)	0.051	0.019	0.006	1.052	1.014–1.092
BMI (kg/m^2^)	0.682	0.128	< 0.001	1.978	1.539–2.544
UA (*μ*mol/L)	0.006	0.002	0.004	1.006	1.002–1.011
TG (mmol/L)	0.910	0.250	< 0.001	2.483	1.520–4.057
HDL‐C (mmol/L)	−0.750	0.475	0.115	0.473	0.186–1.199
HOMA‐IR	0.154	0.074	0.036	1.167	1.010–1.349
lnGDF15	1.925	0.392	< 0.001	6.856	3.180–14.781

*Note:* Logistic regression models were used to explore independent predictors for MS in T2DM patients. The dependent variable was “MS (*yes* = 1, *no* = 0),” and the independent variables were the statistically significant indexes in Table 1.

Abbreviations: BMI, body mass index; DBP, diastolic blood pressure; GDF15, growth differentiation factor 15; HDL‐C, high‐density lipoprotein cholesterol; HOMA‐IR, insulin resistance index; SBP, systolic blood pressure; TG, triglyceride; UA, uric acid.

**Table 5:** Binary logistic regression analysis of factors influencing metabolic syndrome in T2DM patients.

	**Model**	**OR**	**95% CI**		**p** **value**
lnGDF15	Crude model	6.856	3.180	14.781	< 0.001
	Model 1 (adjusted for gender, SBP, and DBP)	6.697	2.898	15.473	< 0.001
	Model 2 (adjusted for gender, SBP, DBP, age, BMI, UA, TG, HDL‐C, and HOMA‐IR)	6.649	2.224	19.885	0.001

*Note:* Binary logistic regression models were used to explore independent predictors for MS in T2DM patients. The dependent variable was “MS (*yes* = 1, *no* = 0)”.

### 4.5. Diagnostic Value of GDF15 for T2DM Combined With MS

**Figure 2:**
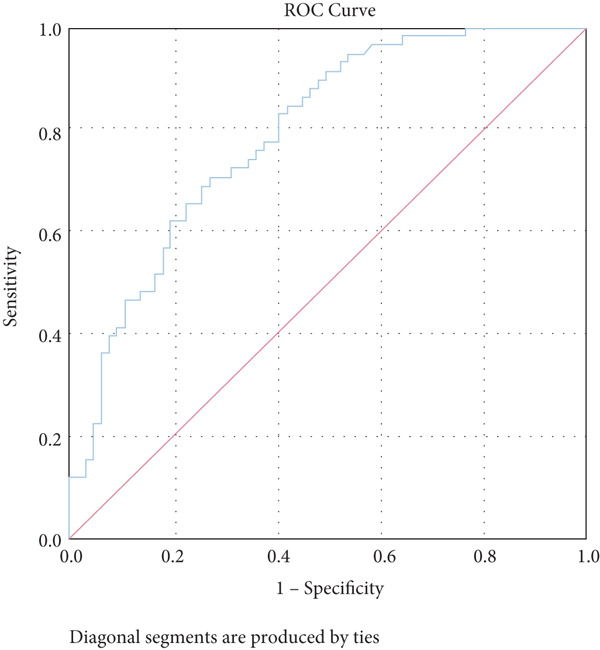
ROC curve of serum GDF15 for diagnosing T2DM combined with MS. Note: The AUC of serum GDF15 for diagnosing T2DM combined with MS was 0.793 (95% CI: 0.716–0.870, *p* < 0.001), with an optimal cut‐off value of 395.81 pg/mL, sensitivity of 0.71, and specificity of 0.73.

The study analysis showed that GDF15 has a certain diagnostic significance for T2DM combined with MS (*p* < 0.05), and the analysis of the ROC curve (Figure 2) showed that the value of the area under the GDF15 curve was 0.793, with an optimal cut‐off value of 395.81 pg/mL, a sensitivity of 0.71, and a specificity of 0.73 (Table 6).

**Table 6:** ROC curve analysis of serum GDF15 for diagnosing T2DM combined with MS.

	**AUC**	**p** **value**	**95% CI**	**Truncation value (pg/mL)**	**Sensitivity**	**Specificity**
GDF15	0.793	< 0.001	0.716–0.870	395.81	0.71	0.73

*Note:* *p* value was significant at 0.05.

## 5. Discussion

MS, also described as syndrome X, IR, and so on, is defined by the WHO as a pathological condition characterized by abdominal obesity, IR, hypertension, and hyperlipidemia [11]. Although this disease has its roots in European and American lifestyle habits, it has become a serious international problem as these behavioral patterns have spread throughout the world. Compared to urban populations in Western countries, the rate of MS is frequently greater among populations of developing countries, especially in metropolitan areas [12]. Proinflammatory, prothrombotic, and progressive, multiple sclerosis can cause a range of disorders, including gout, fatty liver, obstructive sleep apnea, and polycystic ovarian syndrome [13]. It can also raise the risk of cancer [14]. It gets harder to intervene and stop the pathological process as the disease develops [13]. Therefore, early diagnosis and prevention of MS become extremely important.

With a 12.4% overall prevalence, T2DM is a common chronic condition in China [15]. Certain studies have shown that MS is more common in people with T2DM [16], and MS may also lead to exacerbation of T2DM [17]. This study discovered that patients with T2DM with MS had a decrease in HDL‐C and increased SBP, DBP, BMI, TG, and HOMA‐IR levels compared to patients with T2DM alone. These findings suggested that T2DM combined with MS patients had more severe abnormalities of glucose and lipid metabolism. Consistent with the present study, a case‐control study with 293 cases revealed that the MS group had significantly greater BMI, SBP, DBP, TG, and HOMA‐IR and lower levels of HDL‐C than the group without MS [18]. Previous studies have suggested [19] that UA may be a marker of MS, which is consistent with the findings that UA levels were higher in the MS group compared to the non‐MS group in this paper. The pathological mechanism of T2DM is complex and includes insulin secretion dysfunction, lipid metabolism disorders, and other factors, which could lead to the accumulation of several risk factors that could result in MS, which could further worsen IR and raise the likelihood of diabetic complications. Thus, the combination of T2DM and MS makes glycemic control difficult [20, 21]. Consequently, the search for markers that can identify IR and lipid metabolism disorders at an early stage can help to diagnose MS and reduce the risk of T2DM combined with MS and even the risk of cancer through early intervention.

Belonging to the TGF*β* family of cytokines, GDF15 was first identified as having a role in inflammatory and stress pathways. Later on, it was revealed to be an appetite suppressant and a possible treatment for weight loss [22–24]. In this study, we discovered that TG levels rose in tandem with GDF15 levels. Additionally, GDF15 showed a strong correlation (*p* < 0.05) with both TGs (*r* = 0.314) and BMI (*r* = 0.239), indicating a close association between GDF15 and obesity. Previous research has discovered that GDF15 levels are significantly positively correlated with obesity‐related parameters and increased TG [9], and Yunni et al. found that BMI, TG increase, and GDF15 level were significantly positively correlated [18], which further validated the findings of this paper. Furthermore, GDF15 and glucose metabolism are intimately associated [9]. Stress‐induced elevation of GDF15 can activate PPAR*β*/*δ*, suppress phosphorylation of ERK1/2, and stop the decrease of phosphorylated AMPK. These actions inhibit the expression of SOCS3 protein, which in turn inhibits insulin signaling, thereby improving IR [25]. GDF15 expression is associated with p53 [26]. In adipose tissue, p53 is activated, which leads to the generation of cytokines that exacerbate IR, diabetes, and inflammation [27]. In this paper, it was found that by comparing different levels of GDF15 in groups, HOMA‐IR was found to increase with the increase of GDF15 level, but the difference between the groups was not statistically significant, analyzing the reason may be attributed to the small number of samples of the data, which can be further enlarged in the future to study the sample size. In a group of studies on obese subjects [28], GDF15 was higher in the diabetic group and correlated with HbA1c and glucose. However, the results of this study did not show a correlation between GDF15 and glucose or HbA1c, and one of the possible reasons for this analysis was the presence of metformin for glycemic control in the enrolled patients. The most widely prescribed drug for T2DM is metformin, which regulates energy balance by raising GDF15 levels [29]. An additional reason, perhaps as a result of the limited number of patients enrolled in the study and the better glycemic control of most of the enrolled patients, which resulted in concentrated and noncomparable values, and further expansion of the sample size may be needed in the future to make the study results more reliable. In addition, this study also found an association between GDF15 and SBP and UA. While the level of GDF15 increased, the level of SBP gradually increased, and GDF15 was positively correlated with SBP (*r* = 0.274) and UA (*r* = 0.182) (*p* < 0.05), which is in line with the fact that GDF15 is associated with cardiovascular disease [30]. Many other studies have shown that GDF15 is closely and positively correlated with UA [31–33]. In conclusion, as GDF15 and glycolipid are intimately linked, GDF15 can be used as a potential biomarker for the development of diabetes and MS. In this investigation, we discovered that the T2DM with MS group′s level of GDF15 was substantially higher than that of the T2DM alone group. Furthermore, the analysis of clinical data comparing various GDF15 levels showed that the prevalence of MS grew progressively as GDF15 levels rose. Further analysis of GDF15 and MS‐related indexes revealed that GDF15 and the prevalence of MS (*r* = 0.506) were positively correlated (*p* < 0.05). Indicators with differences in the comparison of clinical data of patients with or without combined MS were selected and analyzed by univariate logistic regression analysis, and the findings indicated that SBP, DBP, BMI, UA, TG, HOMA‐IR, and lnGDF15 were the risk factors for T2DM combined with MS and that after adjusting for the composite variables of gender, SBP, and DBP, the risk of MS prevalence increased 6.697‐fold for every one standard deviation change in lnGDF15, and the risk of MS prevalence remained significant after continuing to include the composite variables of age, BMI, UA, TG, HDL‐C, and HOMA‐IR, suggesting that GDF15 is an independent risk factor for MS in the combination of T2DM. Further ROC curve plotting revealed that when GDF15 was greater than 395.81 pg/mL, patients with T2DM had a higher risk of MS.

## 6. Conclusion

In summary, as GDF15 levels grow, those with Type 2 diabetes are more likely to acquire MS. Therefore, serum GDF15 may be a serum marker for metabolic disorders in patients with T2DM combined with MS. Due to the cross‐sectional design and small sample size of the present study, future prospective studies with increased sample size are needed to fully understand the causal relationship between serum GDF15 levels and T2DM and MS.

**Nomenclature**
DM: diabetes mellitus
T2DM: Type 2 diabetes mellitus
MS: metabolic syndrome
GDF15: growth differentiation factor 15
ANOVA: analysis of variance
SBP: systolic blood pressure
DBP: diastolic blood pressure
BMI: body mass index
UA: uric acid
SCR: serum creatinine
TG: triglyceride
CHOL: cholesterol
HDL‐C: high‐density lipoprotein cholesterol
LDL‐C: low‐density lipoprotein cholesterol
CRP: C‐reactive protein
HbA1c: glycosylated hemoglobin A1c
FBG: fasting blood glucose
FINS: fasting insulin
HOMA‐IR: homeostasis model assessment–insulin resistance
CIs: confidence intervals
ORs: odds ratios
ROC: receiver operating characteristic
AUC: area under curve
WHO: World Health Organization
PPAR*β*/*δ*
: peroxisome proliferator–activated receptor‐*β*/*δ*
ERK1/2: extracellular signal–regulated kinases 1/2
AMPK: adenosine 5 ^′^‐monophosphate (AMP)–activated protein kinase
SOCS3: suppressor of cytokine signaling 3

## Ethics Statement

The study conforms to the principles of the Helsinki Declaration, and it was approved by the Clinical Trial Ethics Committee of Hefei Second People′s Hospital (No. 2024‐keyan‐047). Each patient signed an informed consent form. Blood samples were obtained from the remaining samples of routine admission tests, ensuring that there was no additional risk to the patients.

## Disclosure

All authors contributed to the interpretation of the data for the work and revised it critically for important intellectual content. All the authors finally approved the manuscript. All authors have read and agreed to the published version of the manuscript.

## Conflicts of Interest

The authors declare no conflicts of interest.

## Author Contributions

Yiran Zhao and Xiaofang Han were responsible for the conception and design of the study. Yiran Zhao and Yue Li were responsible for data collection. Yiran Zhao was involved in the processing and statistical analysis of data. Yiran Zhao and Xiaofang Han were involved in the drafting of the manuscript. Yiran Zhao and Yonghong Cao contributed equally to this work.

## Funding

This study was funded by the Medical Application Program of the Hefei Municipal Health Commission (Hwk2023zd003).

## Data Availability

The data that support the findings of this study are available on request from the corresponding author. The data are not publicly available due to privacy or ethical restrictions.
